# Assessment of Cortisol, Halitosis and Oral Health in Institutionalized and Non-institutionalized Elderly People

**DOI:** 10.1093/geroni/igaf122.2821

**Published:** 2025-12-31

**Authors:** Fátima Cristina, Carneiro Marques, Élcio Magdalena Giovani, Ivana Barbosa Suffredini

**Affiliations:** UNIP, São Paulo, Sao Paulo, Brazil; UNIP, São Paulo, Sao Paulo, Brazil; UNIP, São Paulo, Sao Paulo, Brazil; UNIP, São Paulo, Sao Paulo, Brazil

## Abstract

**Objective:**

This study aims to evaluate the perception of quality of life of institutionalized elderly people (MB group) in comparison with non-institutionalized elderly people (CLIN group), in addition to comparing these results with dental indices.

**Study Design:**

Both men and women aged 60 years and older were included in the study. Anthropometric and cardiac measurements were taken, in addition to applying specific questionnaires: the GOHAI, aimed at geriatric oral health, and the SF-36, which investigates quality of life.

**Results:**

The Mann-Whitney test (α < 0.05) was used for statistical analysis. No statistically significant differences were observed in the GOHAI domains. Despite the intensive care offered by the institution, non-institutionalized participants reported a better physical quality of life.

**Conclusion:**

The participants presented similar overall oral health conditions. The institutionalized participantsshowed a higher salivary flow. Non-institutionalized participants showed higher salivary cortisollevels. Halitosis proved to be similar. The participants in both groups predominantly used totalmaxillary prostheses, but those in the non-institutionalized group used more partial mandibularprostheses. No differences were found in self-perceived health among the participants. The non-institutionalized participants presented better physical health; however, mental health was similarbetween the two groups.

